# Measurement properties of UCLA Activity Scale for hip and knee arthroplasty patients and translation and cultural adaptation into Danish

**DOI:** 10.1080/17453674.2021.1977533

**Published:** 2021-09-17

**Authors:** Anne Mørup-Petersen, Søren T Skou, Christina E Holm, Paetur M Holm, Claus Varnum, Michael R Krogsgaard, Mogens Laursen, Anders Odgaard

**Affiliations:** a Department of Orthopedic Surgery, University of Copenhagen, Herlev and Gentofte Hospital, Copenhagen;; b Research Unit for Musculoskeletal Function and Physiotherapy, Department of Sports Science and Clinical Biomechanics, University of Southern Denmark, Odense; Research unit PROgrez, Department of Physiotherapy and Occupational Therapy, Naestved-Slagelse-Ringsted Hospitals, Region Zealand;; c Department of Orthopedic Surgery, Copenhagen University Hospital, Rigshospitalet;; d Department of Orthopedic Surgery, Lillebaelt Hospital—Vejle, University Hospital of Southern Denmark; Department of Regional Health Research, Faculty of Health Science, University of Southern Denmark;; e Section for Sports Traumatology, Bispebjerg and Frederiksberg Hospital, Copenhagen, University of Copenhagen;; f Department of Orthopedic Surgery, Aalborg University Hospital, Aalborg & Farsø;;; g Department of Orthopaedic Surgery, Rigshospitalet, Copenhagen University Hospital, Copenhagen, Denmark; Department of Clinical Medicine, University of Copenhagen, Copenhagen, Denmark

## Abstract

Background and purpose — The UCLA Activity Scale (UCLA) is a questionnaire assessing physical activity level from 1 (low) to 10 (high) in patients undergoing hip or knee arthroplasty (HA/KA). After translation and cultural adaptation, we evaluated the measurement properties of the Danish UCLA.

Patients and methods — After dual panel translation, cognitive interviews were performed among 55 HA/KA patients. An orthopedic surgeon and a physiotherapist estimated UCLA scores for 80 KA patients based on short interviews. Measurement properties were evaluated in 130 HA and 134 KA patients preoperatively and 1-year postoperatively.

Results — To suit Danish patients of today, several adaptations were required. Prior to interviews, 4 patients were excluded, and 11 misinterpreted the answer options. Examiners rated the remaining 65 patients (mean age 67 years) 0.2–1.6 UCLA levels lower than patients themselves. The 130 HA and 134 KA patients (mean age 71/68 years) changed from 4.3 (SD 1.9)/4.5 (1.8) preoperatively to 6.6 (1.8)/6.2 (1.0) at 1-year follow-up. 103 (79%) HA and 89 (66%) KA patients reported increased activity. Effect sizes were large (1.2/0.96). Knee patients reaching minimal important change (MIC, ≥ 8 Oxford Knee Score points) had higher 1-year UCLA scores than patients not reaching MIC.

Interpretation — Original scale development was undocumented. Content validity was questionable, and there was discrepancy between patient and examiner estimates. UCLA appears valuable for measuring change in self-reported physical activity on a group level. 4 out of 5 HA patients and 2 out of 3 KA patients were more physically active 1 year after joint replacement surgery.

Hip and knee osteoarthritis (OA) strongly affect a person’s ability to be physically active (Price et al. 2018). When pain and functional impairment becomes so severe that joint replacement is considered, it is of interest for both patients and healthcare providers to know to what degree surgery can be expected to improve a patient’s opportunity to lead an active life. Yet, quantifying physical activity is complex. Accelerometers are often considered as the gold standard for measuring non-specific physical activity; however, being resource demanding they are often not a feasible option and, also, accelerometer results do not necessarily reflect the difficulty of the activities or how important an activity is to each patient (Shephard 2003). As an alternative, physical activity can be quantified using physical activity scales such as the UCLA Activity Scale (UCLA) from University of California, Los Angeles (Table 1) (Amstutz et al. 1984, Zahiri et al. 1998). UCLA is a single-item 10-level-scale, ranging from level 10, representing a highly physically active patient, to level 1, a patient who is dependent on others and unable to leave home.

**Table 1.** UCLA Activity Scale as first published by Amstutz et al. (1984)

Activity level
1Wholly inactive: dependent on others; cannot leave residence
2Mostly inactive: very restricted to minimum activities of daily living
3Sometimes participates in mild activities such as walking, limited housework, and limited shopping
4Regularly participates in mild activities
5Sometimes participates in moderate activities such as swimming and can do unlimited housework or shopping
6Regularly participates in moderate activities
7Regularly participates in active events such as bicycling
8Regularly participates in very active events such as bowling or golf
9Sometimes participates in impact sports such as jogging, tennis, skiing, acrobatics, ballet, heavy labor, or backpacking
10Regularly participates in impact sports

In the later version (Zahiri et al. 1998), level 10 was presented first and a patient instruction was added: “Of the following options, which statement best describes your activity level?”

A description of the development process leading to UCLA has to our knowledge never been published (Amstutz et al. 1984). Originally, it appears to have been made for surgeons to assess activity levels of hip and knee arthroplasty patients (Zahiri et al. 1998). Today, UCLA is used as a patient-reported outcome measure (PROM), although it was probably not developed as such. A study comparing UCLA scores with accelerometer measurements of walking activity revealed a strong correlation but large measurement errors; patients reporting the same level of activity in UCLA varied up to a factor of 15 in average number of steps per day (Zahiri et al. 1998). Despite this, UCLA is widely used internationally and has been recommended as a useful physical activity PROM instruments in hip and knee arthroplasty (HA/KA) patient populations, mainly based on a positive rating of construct validity and high completion rates (Naal et al. 2009, Terwee et al. 2011, Rolfson et al. 2016). Its brevity and simplicity make it an attractive choice, especially when combined with other questionnaires.

In Denmark, UCLA has been used in at least 2 different, unpublished versions. With a direct translation, cultural differences in bicycling habits led to a bimodal distribution of answers (Skou and Roos 2014). This study develops a Danish version of the UCLA through formal translation and cultural adaptation, and further tests the validity, reliability, and responsiveness of the translated questionnaire in relevant groups of hip and knee OA patients before and/or after arthroplasty.

## Patients and methods

**Figure 1.**
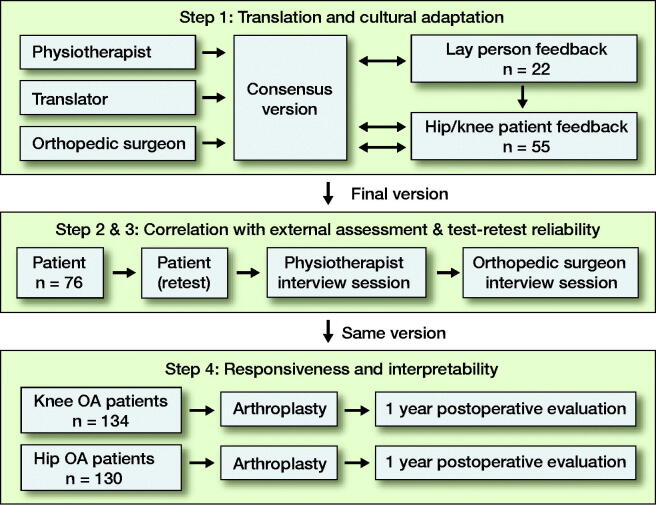
Study overview.

The study was conducted in 4 parts (Figure 1): (1) translation and cultural adaptation, followed by evaluation of (2) correlation with external assessment (by healthcare professionals), and (3) test–retest reliability in KA patients, and (4) construct validity and responsiveness in a cohort of KA and HA patients.

Study design was guided by the COnsensus-based Standards for the selection of health Measurement INstruments (COSMIN) guidelines (Mokkink et al. 2018) and the Guidelines for Reporting Reliability and Agreement Studies (GRRAS) (Kottner et al. 2011).

### Translation and cultural adaptation

A dual panel translation (Swaine-Verdier et al. 2004, McKenna and Doward 2005, Epstein et al. 2015) was made by a professional translator, a physiotherapist, and an orthopedic surgeon (senior house officer, AM) in collaboration; all 3 were English–Danish bilingual with Danish mother-tongue. Each of the three prepared a Danish translation from the original American version (Table 1) and, subsequently, they met to discuss and agree on a consensus version. In case of disagreement, majority ruled.

To ensure wording and cultural adaption, the questionnaire was presented to 3 different laymen panels of total 22 (10 males) heart and lung patients with a mean age of 72 years (SD 9), recruited at physiotherapy team training sessions. Participants completed the questionnaire while “thinking out loud,” and they commented on the questionnaire in plenary sessions. In turn, changes were made in layout, instructions, and activity examples. Subsequently, the revised version was presented in the same manner to target patients: 55 HA or KA patients (38 pre- and 17 postoperative [21 males]), mean age 70 years (SD 8) at Copenhagen University Hospital Gentofte. After 8 rounds of adjustments, the evaluations led to no further revisions and the development process was ended.

### Correlation with external assessment

As UCLA was originally completed by surgeons, we intended to determine the degree of common understanding of activity levels between knee patients and healthcare professionals. During a 3-month period, KA patients (> 40 years, pre- and postoperative, primary and revision KA) were recruited at Naestved Hospital, Region Zealand. We excluded patients unable to read and understand the Danish language and patients with signs of dementia who failed a clock-drawing test (Mainland et al. 2014). Patients filled out UCLA without any help except for a short, written instruction, asking them to consider their physical activity level in the preceding 4 weeks. Age, sex, height, weight, and today’s knee pain level on a visual analogue scale (VAS 0–10, 0 “no pain,” 10 “worst pain imaginable”) was registered. Shortly after, patients were interviewed about daily physical activities for about 5 minutes by (1) a junior orthopedic surgeon (AM/CH) and (2) a physiotherapist (PH), separately and in random order. Each examiner then estimated the patient’s UCLA level (patients were instructed not to reveal their own reported UCLA score).

### Test–retest reliability

After interviews, participants were given a prepaid envelope containing an extra UCLA questionnaire to complete 7–10 days later. On the front page, patients were asked if their physical activity habits had changed since the first test, since only those with unchanged habits were eligible for analysis. Patients awaiting or recovering from surgery (< 6 weeks) were excluded from retests as their activity level was expected to change rapidly.

### Construct validity and responsiveness

UCLA score distribution and responsiveness (validity of change scores) were evaluated at Lillebaelt Hospital—Vejle, Region of Southern Denmark. As part of the normal clinical routine, through 5 months (inclusion March–July 2018), hip OA patients scheduled for HA and knee OA patients scheduled for total or medial unicompartmental KA completed electronic PROM questionnaires (forcing patients to choose only one answer option) before and 1 year after surgery (Procordo Software, Copenhagen). Patients who had revision surgery during year 1 were excluded. Paper versions were available for patients with no email address. Non-responders were reminded by mail and, if necessary, by phone. The PROM questionnaires included UCLA, and the well-established Oxford Hip or Knee Score (OHS, OKS) (Dawson et al. 1996, 1998, Murray et al. 2007, Hossain et al. 2015), the generic EQ-5D-5L and EQ-5D VAS (Jin et al. 2019), and an overall patient satisfaction question (“How satisfied are you with your hip/knee 1 year after surgery?”, 5 answer options, 1 neutral).

Responsiveness was evaluated by use of the construct approach (de Vet et al. 2011), i.e., correlation of UCLA change with other PROM change scores and overall satisfaction. We expected only fair to moderate correlations (Naal et al. 2009) because (1) generic and joint-specific PROMs evaluate factors other than physical activity, (2) joint replacement may improve the ability to be physically active without changing the patients’ habits (because of, e.g., lack of motivation), and (3) perception of change may be influenced by preoperative expectations.

A mean increase of 1–3 UCLA levels 1 year after surgery was expected (SooHoo et al. 2015, Ghomrawi et al. 2017, Scott et al. 2017), as was a 2-fold increase in the proportion of patients with UCLA score ≥ 6 (Scott et al. 2017). We also calculated the effect size, a traditional distribution-based measure to quantify responsiveness (Angst 2011).

### Statistics

UCLA scores were not expected to be equidistant or normally distributed, thus scores were treated as ordinal variables and analyzed using nonparametric statistical methods (Wilcoxon rank sum and Kruskal–Wallis test). To illustrate variations in results, means and standard deviations (SD) were reported as well, and (multiple) linear regression analyses were performed to check for score dependence on age, sex, and BMI. Paired tests (Wilcoxon signed rank test) were used to calculate within-patient differences in UCLA scores. Associations between 1-year (change) UCLA and reaching minimal important change (MIC) of 8 OHS/OKS points were assessed (Beard et al. 2015, Ingelsrud et al. 2018).

For correlation with external assessment, agreements were estimated by mean difference, limits of agreement (LoA), weighted kappa coefficient (Landis and Koch 1977), and Spearman’s correlation coefficient (“very weak” [0–0.19], “weak” [0.20–0.39], “moderate” [0.40–0.59], “strong” [0.60–0.79], and “very strong” [0.80–1.0]).

Floor or ceiling effects were considered present if more than 15% of patients marked the lowest or highest score, respectively (Terwee et al. 2007) and effect sizes were calculated (mean UCLA change/SD_baseline_) (Angst 2011, de Vet et al. 2011). Sample size was based on general recommendations (Terwee et al. 2007, de Vet et al. 2011). Statistical significance level was set at alpha level 0.05 (2-sided), and 95% confidence intervals (CI) were reported when relevant. Analyses were conducted in R (Rstudio) (RCoreTeam; R Foundation for Statistical Computing, Vienna, Austria).

### Ethics, data sharing, funding, and potential conflicts of interest

The study was ethically approved by the National Committee of Health Research Ethics (Jr. no. 16030260). Data management was approved by the Danish Data Protection Agency (Jr. no. 2012-58-0004). Raw data is available upon request. The study was funded by the Health Research Fund of the Capital Region of Denmark. No authors had relevant conflicts of interest.

## Results

### Translation and cultural adaptation

The original American version of UCLA needed comprehensive changes in the aspiration of becoming a valid and patient-relevant measure of physical activity in Danish hip and knee replacement patients of today. For example, bicycling for transportation is very common in Denmark, even among the elderly. To prevent a bimodal score distribution (maximum at levels 4 and 7) as seen in a study based on a previous version (Skou and Roos 2014), the bicycling activity was split by intensity and frequency to cover levels 5–8 in the current version (appendix A [Danish] and B [English translation], see Supplementary data). Acrobatics, ballet, and bowling were exchanged for popular Danish activities, e.g., badminton and gymnastics/fitness. Examples were mentioned only once, but curly brackets illustrated how examples referred to two levels differing by frequency (“regularly” or “once in a while”). The questionnaire had to be self-explanatory, thus a short introductory text was added. During development, we found that thorough instructions led to patients skipping the introduction and misinterpreting the scale and marking multiple boxes or writing numbers instead. Cutting down instructions to a minimum led to fewer misunderstandings.

### Correlation with external assessment

**Figure 2.**
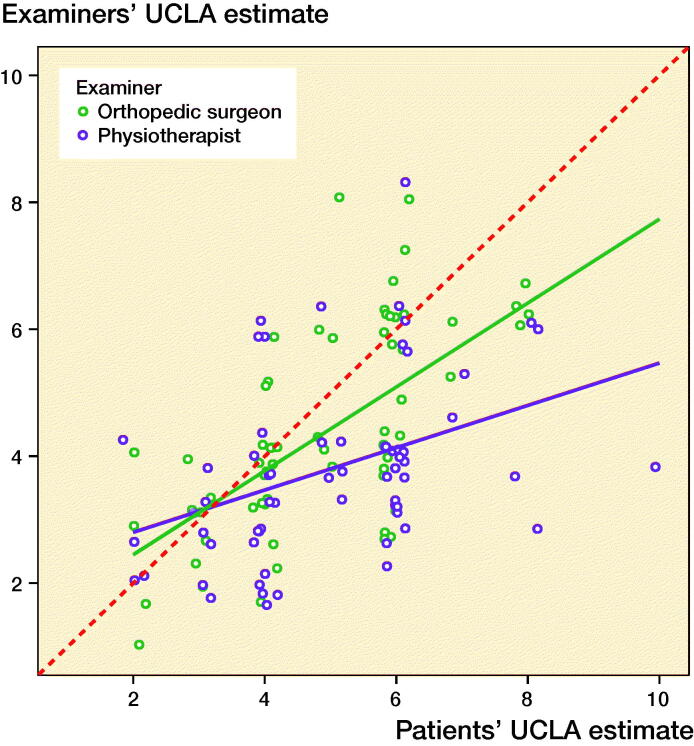
Correlation with external assessment of physical activity level: orthopedic surgeons’ and physiotherapists’ estimates of UCLA plotted against patients’ own estimates, with corresponding regression lines. Random variance (jitter) is added to prevent over-plotting. The red dotted line indicates perfect agreement.

**Figure 3.**
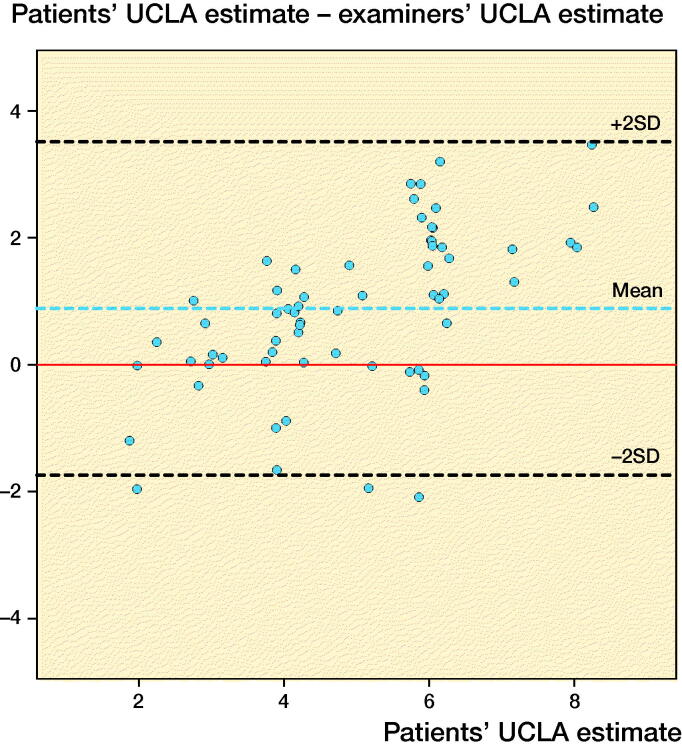
Patients’ UCLA estimate plotted against the difference between patients’ and examiners’ assessments (patient score minus mean of surgeons’ and physiotherapists’ scores) (modified Bland–Altman plot). The dotted lines indicate mean difference (blue) ±2 SD (limits of agreement, black) and hypothetical perfect agreement (red). Random variance (jitter) is added to prevent over-plotting.

We invited 80 knee OA and KA patients for interviews. 2 were excluded due to poor language skills, and 2 because their paper questionnaires were lost. Of 76 patients, 11 (67 years) were excluded because they marked more than 1 answer. The remaining 65 patients (Table 2) overall rated their UCLA level higher than the examiners did (Table 3, Figure 2) and differences increased with UCLA level (Figure 3). In 32 cases, 1 or both examiners agreed perfectly with the patient or the patient’s score was between examiner estimates. The reliability of examiner assessment of patient activity level (Table 3) was “substantial” for surgeons and “fair” for the physiotherapist (Landis and Koch 1977). The corresponding correlations were “strong” and “moderate,” respectively.

**Table 2.** Characteristics of interview participants

Patients (knee arthroplasty/knee OA), n	65 (34/31)
Age, mean [median] (SD)	66 [67] (11)
Male sex, n	29
BMI, mean (SD) (n = 60)	30.4 (5.8)
Pain (VAS 0–10), mean [median] (SD) (n = 63)	4.5 [5] (2.8)

**Table 3.** Results of interviews: correlation with external assessment of physical activity level

UCLA Activity scale			
Absolute scores (total sample)			
	Mean (SD)	Median [IQR]	Range
Patient	5.0 (1.7)	5 [4–6]	2–10
Surgeon	4.4 (1.6)	4 [3–6]	1–8
Physiotherapist	3.8 (1.3)	4 [3–4]	2–8
Differences (within-patient)			
Patient minus	Mean (CI)	Median	Range
surgeon	0.6 (0.2–0.9)	0	–3 to 3
physiotherapist	1.2 (0.8–1.6)	1	–2 to 6
	Reliability	Agreement	Correlation
Patient minus	weighted Kappa	LoA	Spearman’s rho
surgeon	0.63	–2.0 to 3.1	0.65
physiotherapist	0.31	–2.0 to 4.4	0.47

Spearman’s rho correlation coefficient (–1 to 1) indicates the degree of linearity between measurement ranks. LoA = limits of agreement (mean ±2 SD). Differences are based on assessments within each patient.

Patient UCLA was 4.8 (SD 1.7) in females and 5.3 (SD 1.6) in males (difference: CI –0.3 to 1.4). No association was observed between patient UCLA and age (–0.008 per year, CI –0.05 to 0.03) or current knee pain (–0.1 per increase in VAS, CI –0.3 to 0.03), but a small, negative association with BMI was detected (–0.08 per BMI unit, CI –0.15 to –0.01). Results were similar with multiple regression analysis. For examiner estimates, none of these factors were independently associated with activity level.

### Test–retest reliability

Retest questionnaires were returned by 43 of 53 patients. Exclusions were made for 2 who had completed the retest form on day 0, 1 who had marked multiple boxes and 2 returning blank forms. Of the remaining 38, 21 reported to have “unchanged exercise habits” after 8.3 days (range 1–25). In this group, 13 had perfect agreement with their initial score, 5 were 1 level apart, and 1 was 2 levels apart.

### Construct validity and responsiveness

**Figure 4.**
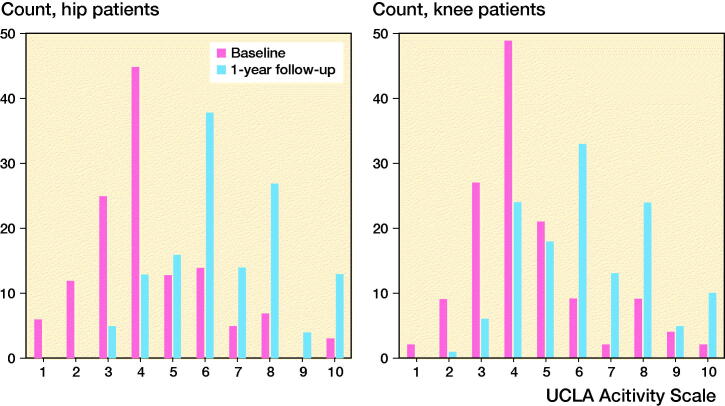
Distribution of pre- and 1-year postoperative UCLA scores in hip (left panel) and knee arthroplasty patients (right panel).

**Figure 5.**
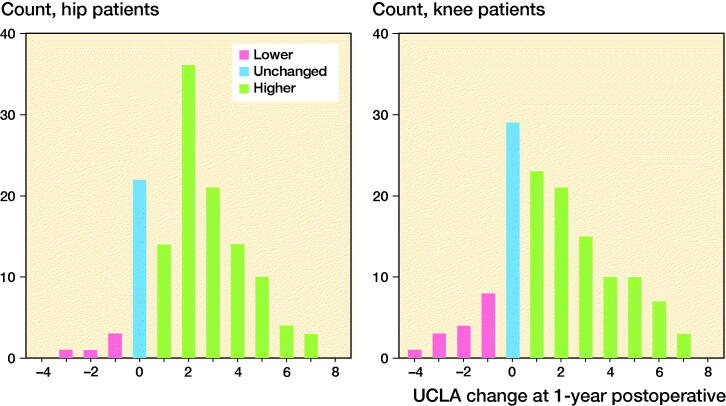
Distribution of UCLA change scores (1-year follow-up minus preoperative score) in hip (left panel) and knee arthroplasty patients (right panel).

Completeness at 1 year reached 96% (HA, n = 130) and 95% (KA, n = 134), respectively. There were no statistically significant sex differences in scores (p = 0.7–1.0). UCLA typically improved from median level 4 to 6 in both groups (Figure 4, Table 4). Positive change in UCLA was reported by 103 (79%) hip and 89 (66%) knee patients (Figure 5). Patient satisfaction and change in other PROMs proved very weak to moderate correlations with UCLA change scores (Table 5), and largest in KA patients. Knee patients reaching MIC (≥ 8 OKS_change_) reported higher 1-year UCLA levels than patients not reaching MIC (1-year UCLA 6.4 and 5.2, respectively, p < 0.04) and had higher change scores (2.1 and –0.2, p < 0.001). In hip patients, the corresponding UCLA scores were 6.6 and 5.7 (p = 0.2) and change scores 2.4 and 1.5 (p = 0.3), respectively. Effect size was 1.2 in HA and 0.96 in KA patients; both “large” (≥ 0.8).

**Table 4.** Patient characteristics and UCLA values in hip and knee arthroplasty patients. Values are count (%) unless otherwise specified

	Patient satisfaction						
Factor	Baseline	1-year postoperative	Very dissatisfied	Dissatisfied	Neither	Satisfied	Very satisfied
Hips	130 (100)	130 (100)	1 (1)	0 (0)	4 (3)	16 (12)	109 (84)
Male/female	62/68 (52/48)	–	0/1	0/0	2/2	9/7	51/58
Age, mean (SD)	71 (9)	–	^b^	–	75 (6)	76 (8)	70 (9)
UCLA ^a^	4.3 [4] (1.9)	6.6 [6] (1.8)	^b^	–	6.3 [7] (2.1)	6.2 [6] (2.3)	6.7 [6] (1.8)
UCLA change ^a^	–	2.3 [2] (2.0)	^b^	–	2.5 [3] (1.9)	1.8 [2] (1.8)	2.4 [2] (2.0)
UCLA ≥ 6	29 (22)	96 (74)	^b^	–	2 (50)	8 (50)	86 (79)
Floor/ceiling ^c^	6/3 (5/2)	0/13 (0/10)	^b^	–	0/0 (0/0)	0/2 (0/13)	0/11 (0/10)
Knees	134 (100)	134 (100)	1 (1)	11 (8)	11 (8)	40 (30)	71 (53)
Male/female	61/73 (46/54)	–	1/0	5/6	6/5	21/19	28/43
Age, mean (SD)	68 (9)	–	^b^	72 (6)	71 (8)	66 (10)	68 (9)
UCLA ^a^	4.5 [4] (1.8)	6.2 [6] (1.0)	^b^	4.6 [4] (1.7)	5.1 [4] (2.2)	6.0 [4] (1.6)	6.8 [4] (1.9)
UCLA change ^a^	–	1.7 [1] (2.3)	^b^	0.0 [0] (1.6)	0.0 [0] (2.0)	1.3 [1] (1.9)	2.6 [2] (2.3)
UCLA ≥ 6	26 (19)	85 (63)	^b^	3 (27)	3 (27)	24 (60)	54 (76)
Floor/ceiling ^c^	2/2 (1/1)	0/10 (0/7)	^b^	0/0 (0/0)	0/1 (0/10)	0/1 (0/3)	0/8 (0/11)

**^a^**Values are mean [median] (SD).

**^b^**Values not shown (1 patient only).

**^c^**Floor/ceiling denotes the number (and percentage) of patients reporting level 1 or 10.

**Table 5.** Change in relevant PROMs grouped by 1-year UCLA improvement. Values are mean (SD) unless otherwise specified

	UCLA improvement					Correlation Spearman’s rho
Factor	≤ –1	0	1–2	3–4	≥ 5	
Hips, n (%)	5 (4)	22 (17)	50 (38)	35 (27)	18 (14)	
1-year OHS	45 (2)	43 (6)	44 (5)	44 (5)	44 (6)	0.09
Δ OHS	24 (5)	20 (7)	22 (8)	25 (8)	27 (9)	0.21
Δ EQ-VAS	13 (26)	20 (15)	24 (27)	33 (22)	38 (27)	0.29
Satisfied or very						
satisfied. n (%)	5 (100)	20 (91)	49 (98)	33 (94)	18 (100)	0.09
Knee, n (%)	16 (12)	29 (22)	44 (33)	25 (19)	20 (15)	–
1-year OKS	34 (10)	38 (9)	38 (9)	39 (5)	45 (4)	0.30
Δ OKS	13 (11)	14 (9)	18 (8)	19 (7)	29 (6)	0.44
Δ EQ-VAS	1 (26)	16 (25)	16 (18)	26 (18)	37 (26)	0.39
Satisfied or very						
satisfied, n (%)	9 (56)	21 (72)	38 (86)	23 (92)	20 (100)	0.39

OHS/OKS: Oxford Hip/Knee Score (0–48, 48 best).

Δ (Delta): change scores from baseline to 1 year postoperatively (EQ-5D-5L results did not provide further valuable information, thus only EQ VAS results are reported). Correlations denote the non-parametrical correlation between the given parameter and UCLA change score (in “satisfaction,” all 5 levels were used in correlation analyses).

## Discussion

The UCLA Activity Scale (UCLA) was translated into Danish with several cultural adaptations required for the scale to be relevant to Danish hip and knee arthroplasty patients of today. Based on interviews, examiners rated patients lower on UCLA than did patients themselves. A 1-year postoperative increase in UCLA was reported by four-fifths of hip and two-thirds of knee arthroplasty patients.

We could not identify reports on the original development and purpose of UCLA. At the time when UCLA was first introduced, there was a need to determine the association between physical activity and polyethylene wear after joint replacement. Since then, polyethylene wear has come to play a smaller role in revisions and the interest in UCLA seems to have shifted towards evaluating the general health benefits of surgery. Despite involvement of patients in the current translation process, the uncertainty of patient involvement in the original scale development remains problematic. UCLA has no proven face or content validity, and this cannot be compensated for by good measurement properties (Mokkink et al. 2018).

Interpretation of UCLA results involves obvious challenges. It encompasses several dimensions in one item: intensity, frequency, activity type, difficulty, and duration. This may be the price paid for brevity, but it can lead to large variations in individual perception of the scale, as the levels are neither mutually exclusive, nor exhaustive. For example, say you work hard at the gym once in a while but you are not able to kneel to do your usual garden work, which activity level should you indicate?

To the best of our knowledge, agreement among patients’ and professionals’ UCLA estimates has not been evaluated before. The systematic differences of 0.2–1.6 points (examiners lower) and wide limits of agreement (95% LoA -2.2–4.4) underline that patient-reported outcomes and professional evaluation are not identical measures, and that interpretation of UCLA may be highly subjective. This, along with previous findings (Zahiri et al. 1998), suggests that comparison of individual patients’ UCLA levels should be made with great caution.

Despite several attempts to make the questionnaire self-explanatory (8 rounds of changes), 11 of 76 patients misunderstood the response options. With an electronic version allowing only 1 response, much of this problem is overcome as patients are guided towards a uniform response to the scale (Gudbergsen et al. 2011). Publications of previous versions of UCLA have not included histograms of score distributions. With the present version, both patients and examiners were more likely to choose levels 4, 6, 8, and 10 (where activities are performed “regularly”) than levels 3, 5, 7 and 9 (performed “sometimes”), perhaps because people tend to have regularity in their life. For example, skiing once a year can be considered a “regular” activity. Theoretically, a more even score distribution might be expected if the term “regularly” were replaced by “often”.

### Interpretation and generalizability

Danish mean scores were comparable to international results: e.g., baseline UCLA in Danish HA/KA patients were 4.3/4.5 corresponding to, e.g., 4.3/4.2 in California (SooHoo et al. 2015). Danish 1-year change from median score 4 to 6 was in accordance with a study of 261 British KA patients (59 years) (Scott et al. 2017). In that study by Scott et al., the number of KA patients reporting that they were very physically active (≥ level 6) increased from 37% to 72% after surgery. In our sample, numbers increased from 26 to 85 of 134 (KA) and from 29 to 96 of 130 (HA), thus the share of very active patients more than tripled in each group. Knee (but not hip) patients with postoperative clinically important improvement in Oxford scores had higher 1-year UCLA and UCLA change scores than others. It is not a given that all patients have a desire to become more physically active after a successful joint replacement, thus, as expected, UCLA correlated only poorly to moderately with other PROMs and overall patient satisfaction where, e.g., pain relief counts, too.

### Limitations and strengths

No previous studies have addressed all measurement property aspects of UCLA or discussed its shortcomings in depth. Regarding reliability, we found no floor or ceiling effect, which was in accordance with previous studies (Naal et al. 2009). However, due to the low retest sample size (21 patients), we are reluctant to calculate weighted kappa or make conclusions about measurement error, an important aspect of reliability, which remains uncertain. The reported (lack of) association between UCLA and age, sex, pain, and BMI should be considered with caution, as the study was not powered to study these matters.

## Conclusion

Based on the findings of this study, the UCLA Activity Scale (UCLA) probably cannot provide a valid measure of physical activity level in the individual patient, but the scale is useful on a group level. 1 year after joint replacement, 4 out of 5 hip patients and 2 out of 3 knee patients were more physically active. This information is relevant to hip and knee osteoarthritis patients considering joint replacement surgery. Authors recommend use of an electronic version of UCLA, if possible. Future reliability studies should include retests of more patients, and responsiveness studies should include a specific anchor question regarding change in physical activity to allow for an anchor-based calculation of minimal important change (MIC_UCLA_). Validation of UCLA against other more comprehensive patient-reported activity scales, accelerometers, or performance-based measures would be interesting, though the underlying construct may be very different across measurement methods.

## Supplementary Material

Supplemental Material

## Supplementary data

Appendices are available as supplementary data in the online version of this article, http://dx.doi.org/10.1080/17453674.2021.1977533
