# Supplementary material for: Measurement properties of UCLA Activity Scale for hip and knee arthroplasty patients and translation and cultural adaptation into Danish
Source: Acta Orthop. 2021 Sep 17;92(6):681–8. doi: 10.1080/17453674.2021.1977533 (PMC8635665; doi:10.1080/17453674.2021.1977533)
Supplement: Supplemental Material [file IORT_A_1977533_SM9730.pdf]

Supplementary data

UCLA Aktivitetsskala

Hvor højt er dit aktivitetsniveau lige nu?

Tænk på dit aktivitetsniveau i de seneste 4 uger.
Niveau 10 er meget højt, og niveau 1 er meget lavt.
Sæt kun ét kryds ved én af de ti muligheder.

Sæt kun ét X

10	☐	**Jeg er regelmæssigt fysisk aktiv med høj belastning**	*f.eks. løb, fodbold, håndbold, badminton, tennis, skiløb, tungt fysisk arbejde, bjergvandring eller lignende*
9	☐	**Jeg er af og til fysisk aktiv med høj belastning**	
8	☐	**Jeg er regelmæssigt meget fysisk aktiv**	*f.eks. lange cykelture, golf, krævende gymnastik/fitness eller lignende*
7	☐	**Jeg er af og til meget fysisk aktiv**	
6	☐	**Jeg er regelmæssigt fysisk aktiv**	*f.eks. svømning, cykelture, lange gåture, krævende husligt arbejde, større indkøb eller lignende*
5	☐	**Jeg er af og til fysisk aktiv**	
4	☐	**Jeg er regelmæssigt lettere fysisk aktiv**	*f.eks. gang, let husligt arbejde, mindre indkøb eller lignende*
3	☐	**Jeg er af og til lettere fysisk aktiv**	
2	☐	**Jeg er nærmest aldrig fysisk aktiv**	*begrænset til et minimum af dagligdags aktiviteter*
1	☐	**Jeg er slet ikke fysisk aktiv**	*afhængig af andre, kan ikke forlade hjemmet*

 Acta Orthopaedica 2021; 92 (DOI: 10.1080/17453674.2021.1977533)

UCLA Activity Scale

How is your level of physical activity at the moment?

Consider your level of physical activity for the past 4 weeks.

Level 10 is very high and level 1 is very low.

Please mark only one of the ten options.

↓ ***Mark one option only with an X***

10	☐	**I regularly do high-impact physical activities**	*e.g., running, football, handball, badminton, tennis, skiing, heavy labor, hiking, etc.*
9	☐	**I sometimes do high-impact physical activities**	
8	☐	**I am regularly very physically active**	*e.g. long bicycle rides, golf, high intensity gym or fitness, etc.*
7	☐	**I am sometimes very physically active**	
6	☐	**I am regularly physically active**	*e.g. swimming, bicycling, long walks, heavy housework, unlimited shopping etc.*
5	☐	**I am sometimes physically active**	
4	☐	**I am regularly mildly physically active**	*e.g., walking, light housework, limited shopping etc.*
3	☐	**I am sometimes mildly physically active**	
2	☐	**I am almost never physically active**	~ *limited to a minimum of daily activities*
1	☐	**I am not at all physically active**	~ *Dependent on others, cannot leave home*

Please note: *This English translation of UCLA is published as a rather direct translation of the validated Danish version of UCLA Activity Scale, 2021. This English version has not been validated or developed among English-speaking patients and authors recommend that any use of the questionnaire is preceded by a thorough translation and validation process.*
